# RIVET: comprehensive graphic user interface for analysis and exploration of genome-wide translatomics data

**DOI:** 10.1186/s12864-018-5166-z

**Published:** 2018-11-08

**Authors:** Amanda W. Ernlund, Robert J. Schneider, Kelly V. Ruggles

**Affiliations:** 1 Department of Microbiology, New York University School of Medicine, New York, 10016 USA; 2 Department of Medicine, Department of Translational Medicine, New York University School of Medicine, New York, 10016 USA

**Keywords:** Ribosome profiling, Polysome profiling, Translational regulation, Translational efficiency, Differential expression, R shiny

## Abstract

**Background:**

Translatomics data, particularly genome-wide ribosome profiling and polysome profiling, provide multiple levels of gene regulatory information that can be used to assess general transcription and translation, as well translational efficiency. The increasing popularity of these techniques has resulted in multiple algorithms to detect translational regulation, typically distributed in the form of command line tools that require a basic level of programming ability. Additionally, due to the static nature of current software, dynamic transcriptional and translational comparative analysis cannot be adequately achieved. In order to streamline hypothesis generation, investigators must have the ability to manipulate and interact with their data in real-time.

**Results:**

To address the lack of integration in current software, we introduce RIVET, Ribosomal Investigation and Visualization to Evaluate Translation, an R shiny based graphical user interface for translatomics data exploration and differential analysis. RIVET can analyze either microarray or RNA sequencing data from polysome profiling and ribosome profiling experiments. RIVET provides multiple choices for statistical analysis as well as integration of transcription, translation, and translational efficiency data analytics and the ability to visualize all results dynamically.

**Conclusions:**

RIVET is a user-friendly tool designed for bench scientists with little to no programming background. RIVET facilitates the data analysis of translatomics data allowing for dynamic generation of results based on user-defined inputs and publication ready visualization. We expect RIVET will allow for scientists to efficiently make more comprehensive data observations that will lead to more robust hypothesis regarding translational regulation.

**Electronic supplementary material:**

The online version of this article (10.1186/s12864-018-5166-z) contains supplementary material, which is available to authorized users.

## Background

Regulation of gene expression is a multi-tiered process critical for maintaining cellular homeostasis. Upon disruption of regulatory mechanisms, gene expression can become disrupted at the level of transcription, cytoplasmic export, RNA stability, RNA localization, and at the level of mRNA translation leading to aberrant protein expression. Translational control of mRNA can be achieved globally, via cellular mechanisms that modulate the levels of protein production from all mRNA, or in a targeted process, through mechanisms that promote or inhibit the specific translation of a subset of mRNAs [1]. Aberrant translation of mRNA has been shown to play an important role in many diseases, particularly in cancers and cancers refractory to treatment [2, 3].

**Fig. 1:**
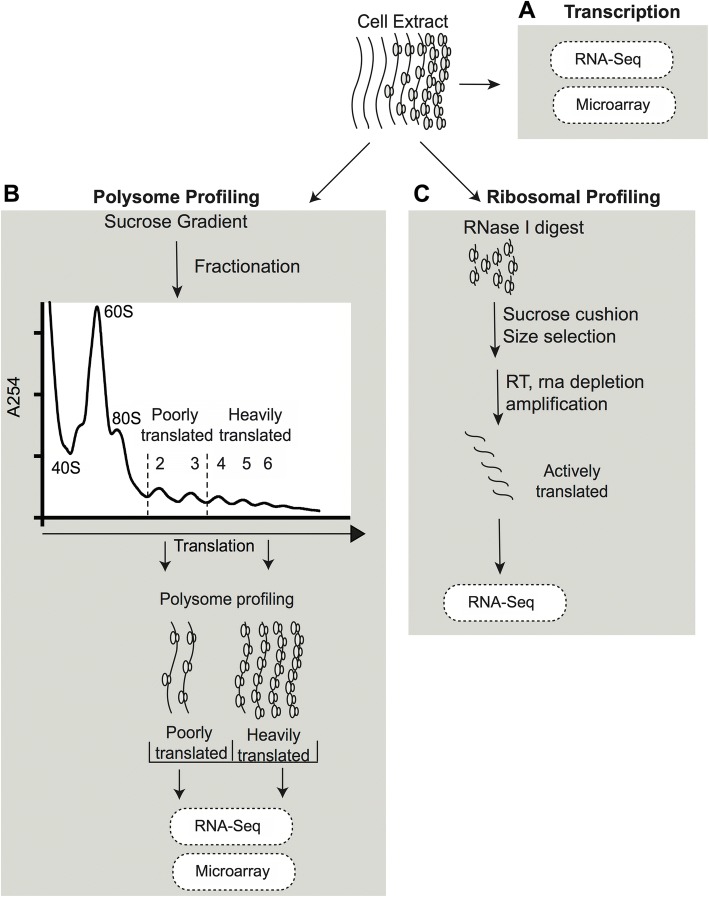
Genome-wide translatome assays. **a**. Schematic of polysome profiling and ribosome profiling. Cytoplasmic mRNA is first isolated and subjected to density ultracentrifugation in both methods where mRNAs bound to ribosomes are separated based on weight and velocity. A sample of cytoplasmic mRNA is maintained for downstream analysis. **b**. In polysome profiling, ribosome-bound mRNAs are further fractionated based on ribosomal RNA content and segregated into fractions based on bound number of ribosomes. Collected fractions of ribosome-bound mRNAs can be pooled based on ribosome number to analyze different levels of translation. The cytoplasmic sample and polysome sample or samples are then subjected to microarray analysis or RNA sequencing. **c**. In ribosome profiling, ribosome-bound mRNAs are subjected to RNase treatment prior to ultracentrifugation, which digests naked mRNA leaving 23–27 nt fragments of ribosome-protected mRNA. These fragments are size-selected and amplified. The cytoplasmic sample and footprint sample are then subjected to RNA sequencing

Two commonly used approaches to study genome-wide translational control are polysome profiling and ribosome profiling (reviewed in [4]). Polysome profiling captures both transcriptional and translational regulation by isolating cytoplasmic RNA and enriching for actively translating mRNA bound to ribosomes. Enrichment of actively translated mRNA is performed by separation of ribosome-bound mRNA using sucrose-gradient ultracentrifugation followed by fractionation of mRNA pertaining to the quantity of bound ribosomes followed by RNA-seq or microarray analysis for transcriptional quantitation (Fig. 1b). Cytoplasmic mRNA preserved prior to ribosome-enrichment as well as polyribosome enriched mRNA fractions are then used to measure active transcription by performing microarray analysis or sequencing on total RNA (Fig. 1a). Thereby, each mRNA in the cell will have measurements for transcriptional changes as well as translational changes occurring due to varied experimental conditions. And, as polysome profiling allows for an exact enrichment of mRNA corresponding to the number of bound ribosomes, changes that shift the level of translation of a particular mRNA can also be examined through density-based segmentation of the polysome fractions [5] (Fig. 1b). An alternate technique, ribosome profiling, also utilizes a multi-omics approach to capture both transcriptional and translational effects, however, exclusively quantifies mRNA using an RNA sequencing platform (Fig. 1c). Additionally, ribosome profiling differs from polysome profiling in that nuclease digestion is performed with ribosome-enriched mRNA, degrading unbound mRNA and leaving a ribosome protected RNA footprint of ~ 30 nts. Nuclease digestion allows for additional analysis of location of the ribosome on mRNA. For differential expression analysis, only a transcription and translational fraction of mRNA can be captured utilizing ribosome profiling [6]. Both techniques are schematized in Fig. 1.

Datasets derived from translational control experiments have the ability to provide multiple layers of information regarding mRNA regulation including transcriptomic and translatomic data. Further, these experiments allow for examination of translation independent from transcription, also known as translational efficiency. Recently, with the upsurge in the use of ribosome profiling, there has been a corresponding increase in algorithms that detect translational efficiency that can be applied to ribosome profiling and RNA sequencing-based polysome profiling data [7–10]. However, these algorithms model discrete data based on negative binomial distributions, and therefore should not be applied to continuous based microarray data, which is prohibitive to the analysis of publicly available genome-wide polysome profiling microarray datasets. Additionally, these packages rarely implement comprehensive data visualization tools and require the use of R-packages and/or command-line interfaces, restricting use to scientists proficient in programming [10]. The inability to easily perform differential analysis and visualization for both polysome and ribosome profiling highlight the need for a user-friendly tool that can adapt to both polysome profiling and ribosome profiling data sets.

Here we present RIVET, an R shiny graphical user interface to identify mRNA regulation occurring in the transcriptome, translatome, and to identify those genes that are regulated at the level of translation independent of transcription. RIVET is a comprehensive user interface (UI) that takes in a user-defined matrix of RNA-seq counts data or microarray data, normalizes the data and performs differential expression analysis with several options for statistical packages, followed by comprehensive data visualization. RIVET is based on limma [11] and edgeR [12] statistical modeling packages and utilizes ggplot2 [13] graphical environment. To perform differential analysis for translational efficiency, the user can choose to utilize a log_2_ ratio of polysome to total [6, 14] or heavier polysome to lighter polysomes [15, 16] or a statistical model incorporating an interaction between condition and polysome to total [17, 18]. RIVET differs from other bioinformatics tools that it provides normalization, differential expression detection at the level of transcription and translation including translation occurring within multiple polyribosome fractions, and publication-ready visualizations. RIVET simplifies the process of analyzing translatome-based genome-wide data allowing users to gain critical insights from data quickly and interactively.

### Implementation

#### Rivet UI architecture

**Fig. 2:**
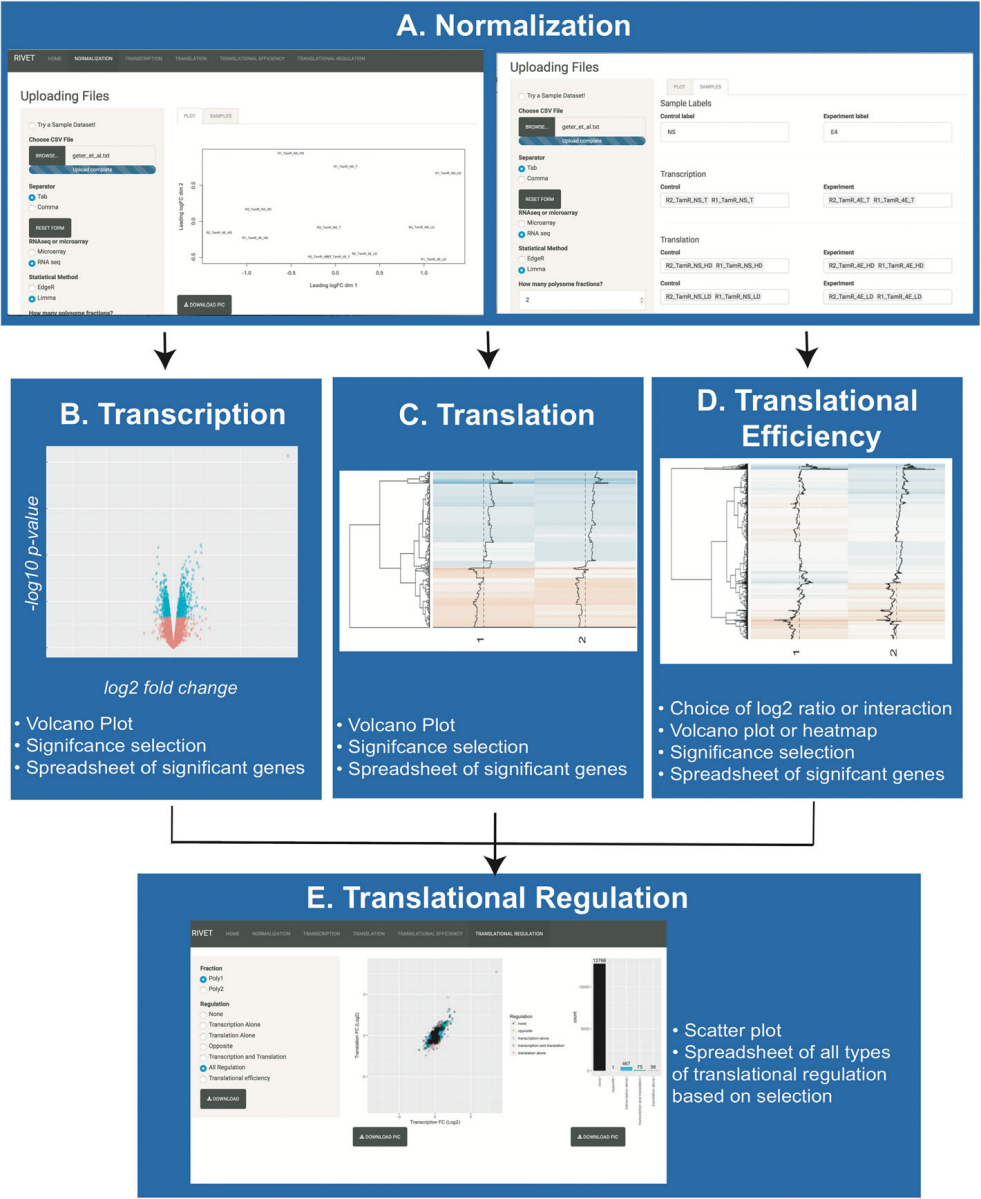
User Interface Architecture. **a**. Flow of Analysis performed. Users will initially upload data matrices and input analysis parameters in the normalization module. Statistical analysis will automatically be performed based on input parameters. Users can then dynamically select regulated-mRNAs in transcription, translation, or translational efficiency modules. The translational regulation module utilizes parameter input from both transcription and translation modules, so should be performed following both transcription and translation module parameter input if default settings are undesired for final analysis. **b**. Module Description. RIVET is divided into 5 modules. The normalization module offers options for statistical parameters as well as MDS plot for quality control and a downloadable spreadsheet of all mRNAs with fold-changes and *p*-values. The transcription module offers a dynamic volcano plot that where the user can select thresholds for fold-change and p-value that will update the plot with colored significant mRNAs. The translation module and translational efficiency module offer a volcano plot or heatmap depending on number of input polysome fractions as well as sliders to select significance thresholds. In addition, the translational efficiency module allows for user to select which method to use to analyze for translational efficiency: log2ratio or interaction. The translational regulation module allows the user to examine types of translational regulation in the form of a scatter plot and barplot. The download spreadsheet feature labels all selected mRNAs with the type of translational regulation. All modules allow for download of visuals as well as statistical analysis download

The RIVET UI (http://ruggleslab.shinyapps.io/RIVET) is comprised of 5 components split into modules: normalization, transcription, polysome, translational efficiency, and translational regulation (Fig. 2). In order to use RIVET, users begin with the normalization module and input a matrix of counts data from RNA sequencing experiments or a background-corrected normalized matrix of microarray data. Based on input data, a multidimensional scaling (MDS) plot is automatically generated for the user to ascertain sample quality and potential batch effects. The user can then select the number of polysome fractions to analyze and segregate samples into control and experimental groups and transcription and translation groups. Following sample segregation, differential expression analysis is automatically performed, using either limma or edgeR, based on user preference (Fig. 2a). Upon completion of all tasks in the normalization module, the user can toggle between the transcription module, the polysome module, the translational efficiency module, or the translational regulation module.

The transcription module focuses on transcription in isolation of translation, generating a volcano plot visualizing effect size of differential analysis at the transcriptional level. The user can choose thresholds to consider for most differentially expressed mRNAs through which a downloadable spreadsheet of selected mRNAs is generated (Fig. 2b). The translation module focuses on translation alone, generating either a volcano plot, in the case of one polysome fraction, or a heatmap in the case of multiple polysome fractions (Fig. 2c). The translational efficiency module takes both transcription and translation into account, generating a scatter plot visualizing different types of translational regulation and a downloadable spreadsheet corresponding to genes regulated in different ways (e.g. transcription alone, transcription and translation, translation alone). The user can choose to analyze translational efficiency using either the log2 ratio method or the interaction term method [17, 18](Fig. 2d). All modules allow for download of user-selected differential expression and dynamically-created visualizations.

#### Data input

Typical polysome and ribosome profiling experiments utilize microarrays or RNA sequencing to quantify both transcriptional data as well as ribosome-enriched data. Data input for RIVET, from microarrays or RNA sequencing, must be in matrix format with samples as columns and genes as rows; most RNA sequencing pipelines and microarray pipeline output data in RIVET ready to use format. Data from microarrays must be background corrected prior to input into RIVET. Most publicly deposited microarray data can be easily downloaded as a matrix of data with background correction and data normalization already performed. Examples of appropriately formatted data can be found in Additional file 1: Table S1 If the user needs to perform background correction and normalization for raw microarray CEL files, we refer the user to the GenePattern suite of graphical user interface tools that can be utilized to generate a spreadsheet of expression values as RIVET input. In brief, to use GenePattern to create RIVET input files, the user should provide a zip archive of Affymetirx CEL files from microarray experiments. If microarray files are derived from 3′ biased IVT Affymetrix arrays, the user can use the ExpressionFileCreator module to upload zip file of arrays. Default parameter settings using RMA background correction and quantile normalization will output a GCT formatted file that can be uploaded to RIVET. If microarray files are derived from Affymetrix 1.1, 2.0, 2.1 ST arrays, exon arrays, HTA 2.0 arrays, the module AffySTExpressionFileCreator can be used with a zip archive of Affymetrix CEL files and default user settings. For more information see the GenePattern software website, genepattern.broadinstitute.org [19].

Because RIVET RNA sequencing-based statistical analyses utilize statistics that assume negative binomial distributions or perform normalization that requires discrete counts data, RNA sequencing data must be input as a raw counts matrix; TPM, RPKM, FPKM normalization cannot be handled by RIVET statistical analysis. Appropriate RNA sequencing counts table are generated by commonly used RNA-seq pipelines including STAR [20] or HTSeq [21].

#### Sample selection and user-defined analysis choices

The normalization module of RIVET is split into a sidebar panel allowing the user to choose multiple parameters for defining statistical analysis and experimental design, as well as a main panel containing information regarding sample categorization and a quality control MDS plot. Matrix-formatted data can be uploaded into RIVET via the sidebar upload feature. When provided with an input counts table, RIVET automatically generates an MDS plot for quality control purposes in the main page under the plot module. Within the main page samples module, RIVET provides the user with an interface to label samples based on experiment and control as well as whether samples were generated from transcriptional or translational data (Additional file 2: Figure S1B). To group samples, the user is presented with drop-down menus containing the names of samples as defined by the column headers in the input matrix and must choose how to categorize each sample according to experiment type. Additionally, when inputting polysome data, the user has the option to define additional translational categories depending on the number of polysome fractions in the experiment. When more than one translational fraction is defined, the software will dynamically generate additional experiment and control input boxes to further categorize polysome samples. For most polysome and ribosome profiling experiments, the default box allowing for input of 1 experiment sample type and 1 control is appropriate. In order to run statistical analysis, the user must complete all prompts in the main panel of the samples module; default values in all other options in the sidebar panel are sufficient to complete the run.

In order to provide an example of user-defined analysis input, we will use a simplified experimental design from Geter et al. [2] (GSE107590). In this study, the authors silenced the expression of eIF4E (experiment) in Tamoxifen-resistant breast cancer cells compared to a non-silencing control (control) using biological duplicates. The authors performed polysome profiling on experiment and control samples where a sample from cytoplasmic RNA (total) was utilized to quantify transcription in each condition. In addition, mRNAs bound to 2–3 ribosomes (light) and 4–6 ribosomes (heavy) in each condition were enriched so that in total there were 12 samples (Additional file 2: Figure S1A). Samples isolated from total mRNA would be entered into the Transcription category with control samples replicate 1 and 2 entered into the control box and experiment samples replicate 1 and 2 entered into the experiment box. The number of polysome fractions can also be changed to automatically allow for additional inputs for associated samples (Additional file 2: Figure S1B).

#### Pipeline architecture

**Fig. 3:**
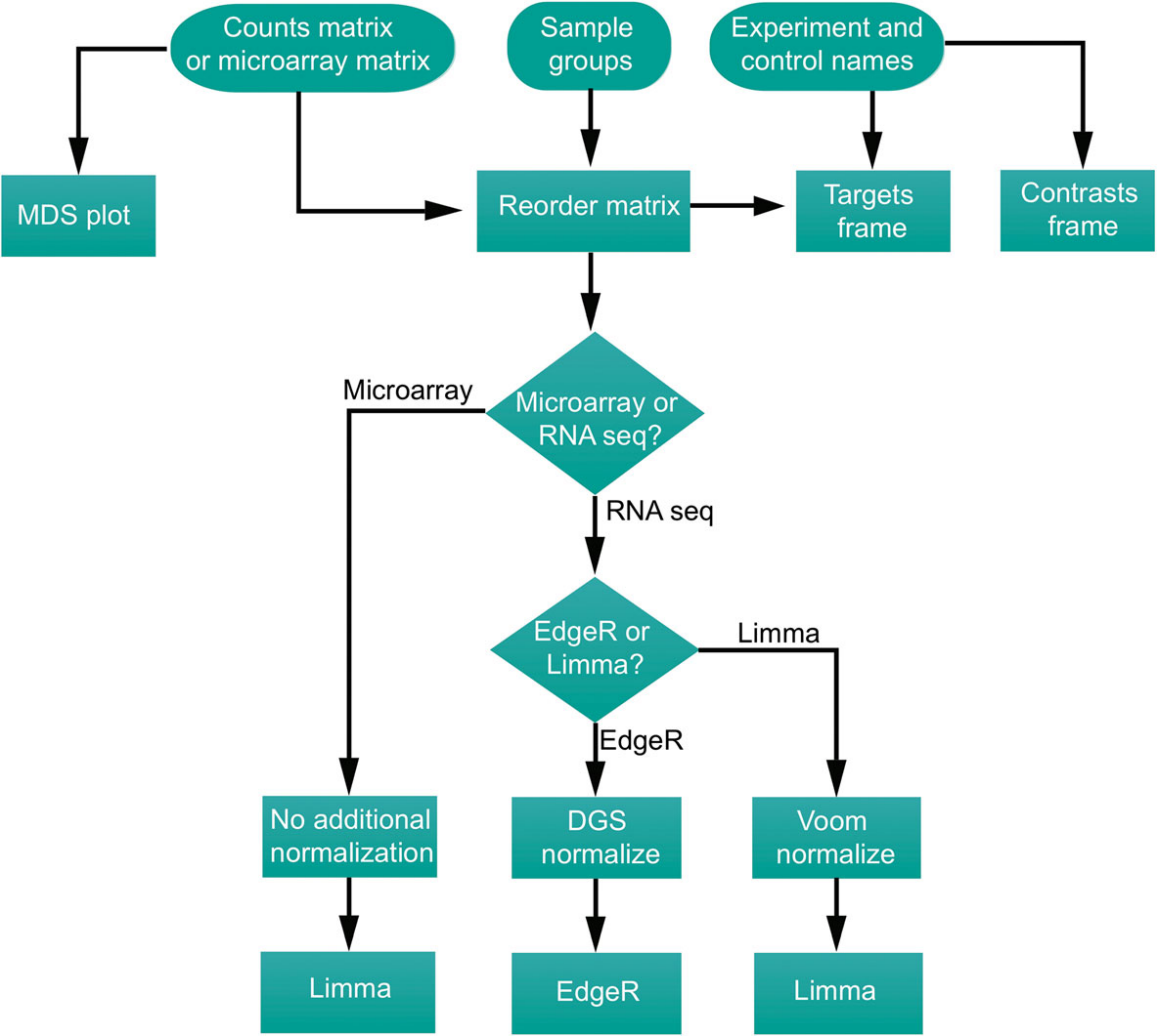
Control flow of statistical analysis performed by RIVET. Flow diagram of user input and RIVET analysis. The RIVET framework takes 3 forms of user input (circles on flow diagram): uploaded normalized microarray matrix or RNA sequencing counts matrix, user-defined control and experiment samples in both transcription and translational fractions, names of the experiment and control groups. Three object types are created using the three user-inputs: 1) counts matrix and sample groups are used to generate a reordered matrix of genes by samples. 2) The reordered matrix and user-defined experiment and control names are used as input to generate a targets dataframe required by both edgeR and limma as part of defining the experimental design. 3) User-defined experiment and control names are used to generate a contrasts dataframe defining the contrasts as experiment-control for translation and transcription samples. Allow flow streams converge on the user selection of microarray vs. RNA sequencing. If microarray, the reordered matrix, targets frame, and contrasts frame are used as input to limma. If RNA sequencing, the user is prompted to select between limma or edgeR statistical analysis choices. If limma is chosen, the reorder-matrix is voom normalized and used as input along with the targets frame and contrasts frame into a limma. If edgeR is chosen, the reordered matrix is DGE normalized and input along with targets frame and contrasts frame into edgeR

Upon input of a matrix of expression values and completion of required parameters, RIVET automatically carries out two work flows simultaneously, the first workflow generates normalized data to be used in the statistical model, the second workflow will generate a targets matrix and contrasts matrix defined based on user input labels for experiment and control, as well as, user input sample groupings (Fig. 3). The first workflow will normalize counts data utilizing the dge object for edgeR statistical analysis or voom normalization (limma package) as indicated by the user. The second work flow will produce a targets dataframe and contrasts dataframe in the following manner: 1) a targets object will be generated based on user defined sample groupings from the main page of the normalization module and the column headings of the matrix of reordered sample labels. The targets object will contain information regarding the assignment of samples to a particular label class. In the example above regarding eIF4E silencing vs control tamoxifen resistant cells, the sample classes would be as follows: *NS.transcription*, *E4.transcription, NS.translation1, E4.translation1, NS.translation2, E4.translation.2*. Samples would be assigned as follows: *NS.transcripion*: replicate 1 control transcription, replicate 2 control transcription, *E4.transcription*: replicate 1 experiment transcription, replicate 2 experiment transcription, *NS.translation1*: replicate 1 control translation heavy polysome, replicate 2 control translation heavy polysome, *4E.translation1*: replicate 1 experiment translation heavy polysome, replicate 2 experiment translation heavy polysome, *NS.translation2*: replicate 1 control translation light polysome, replicate 2 control translation light polysome, *4E.translation2*: replicate 1 experiment translation light polysome, replicate 2 experiment translation light polysome (Table 1) 2) In tandem, a contrasts matrix will be defined based on user-defined labels of control and experiment in the samples submodule of the normalization module. The contrasts matrix for the above example would compare the means for 1) NS vs 4E transcripton, 2) NS vs 4E translation heavy, 3) NS vs 4E translation light (Table 2). Statistical analysis will utilize normalized data, the targets frame, and contrasts frame as input.

**Table 1:** RIVET-Generated Targets Frame

	NS.transcription	E4.transcription	NS.translation.1	E4.translation.1	NS.translation.2	E4.translation.2
R1_NS_TamR_T	1	0	0	0	0	0
R2_NS_TamR_T	1	0	0	0	0	0
R1_4E_TamR_T	0	1	0	0	0	0
R2_4E_TamR_T	0	1	0	0	0	0
R1_NS_TamR_H	0	0	1	0	0	0
R2_NS_TamR_H	0	0	1	0	0	0
R1_4E_TamR_H	0	0	0	1	0	0
R2_4E_TamR_H	0	0	0	1	0	0
R1_NS_TamR_L	0	0	0	0	1	0
R2_NS_TamR_L	0	0	0	0	1	0
R1_4E_TamR_L	0	0	0	0	0	1
R2_4E_TamR_L	0	0	0	0	0	1

**Table 2:** RIVET-Generated Contrasts Frame

	E4.transcription-NS.transcription	E4.translation.1-NS.translation.1	E4.translation.2-NS.translation.2
NS.transcription	−1	0	0
E4.transcription	1	0	0
NS.translation.1	0	−1	0
E4.translation.1	0	1	0
NS.translation.2	0	0	−1
E4.translation.2	0	0	1

For generation of translational efficiency data, user input data will be used in a similar manner but contrasts frame along with statistical model will be different. The statistical model will be defined as follows:

~ 0 + type + treatment + type:treatment.

In the above statistical model, type defines polysome or cytosolic RNA and treatment defines the experimental conditions tested. The contrasts matrix will define translational efficiency as the interaction of type and treatment. If the user chooses to utilize log_2_ ratio, a matrix of ratios will be calculated grouped by [polysome/total]_control_ and [polysome/total]_experiment._ The matrix will then be used as input to normalization and contrasts matrix will be defined as previous.

Upon completion of statistical analysis, a matrix is generated containing the log_2_ fold-change for experiment standardized to control of every mRNA, as well as *p*-values and adjusted p-values for all comparisons. These statistics are generated for both transcription and for each polysome fraction within this matrix and is available for download as a tab-delimited text file at the bottom of the normalization sidebar menu.

#### Transcription and translational analysis

As the full statistical analysis results are provided in the normalization module, the transcription and translation modules allow users to define a threshold of significance for differential analysis in real time, with complementary dynamic visualization of data based on the threshold criteria chosen. Within the transcription module, users use sliders provided in the sidebar panel to control the threshold of log fold-change, *p*-value cutoff, or adjusted p-value cutoff. A volcano plot is generated in the main panel automatically with default settings of > 1 log-fold change and *p*-value < 0.05. Based on user-defined thresholds, the number of significant genes, highlighted in blue on the plot, will automatically adjust (Fig. 2b). Once the user is satisfied with threshold criteria, a tab-delimited text file of selected genes with log-fold change, p-value, and adjusted p-value can be downloaded.

The user interface layout of the translation module is similar to that of transcription with slider options to choose log fold change and p-value / adjusted p-value significance thresholds. If provided with one translational fraction, a volcano plot is also generated displaying user-defined significant genes. However, if more than one polysome fraction is provided, the list of significant genes per each fraction is concatenated and the log-fold change of each mRNA across polysome fractions is displayed as a heatmap (Fig. 2c). In this way, the user can view the dynamics of each mRNA’s log-fold change across polysome fractions to ascertain modulations in the level of translation of each mRNA due to treatment. Both heatmap and text file can be downloaded of this analysis.

#### Translational efficiency analysis

Within the translational efficiency module, the user can utilize the sidebar menu to choose what method to determine translation efficiency: log_2_ ratio or interaction (defined above). Thresholds for fold-change as well as *p*-value or adjusted p-value are included in this panel. The main panel includes either a volcano plot for a single polysome fraction or heatmap for multiple polysome fractions. When the user selects a log-fold change threshold, the log-fold change refers to the ratio of polysome/total_experiment_ vs. polysome/total_control._ The visualization of multiple polysome fractions for translational efficiency, displayed as a heatmap, is the log fold change of polysome_experiment_ and polysome_control_ as translational efficiency is a way to select for mRNAs with greater change in translation than transcription, however, the fold-change in polysomes is more biologically relevant_._ The genes displayed in the heatmap are those that are defined as having significant differences in translational efficiency due to treatment (Fig. 2d).

#### Examination of types of translational regulation

A variety of biological mechanisms underlie translational regulation. Comparing mRNA expression levels in transcription and translation can help characterize this regulation. These types of regulation include translational buffering, where changes in mRNA expression at the level of transcription are not reflected in translation; changes in transcription that are directly reflected in translation; changes occurring that are opposite in magnitude in transcription and translation; and lastly, changes occurring at the level of translation and not transcription.

User defined thresholds for significance at the transcription and translation level are used to determine the category of regulation assigned to each mRNA in the translational regulation module. If the user chooses to navigate to the translational regulation module directly following completion of the normalization tab, the module will use the default thresholds (*p*-value < 0.05) to select mRNAs to highlight on the scatter plot. Our tool allows users to toggle between the different types of translational regulation: transcription alone, translation alone, transcription and translation, and opposite. This provides users the ability to view mRNAs that are regulated in all types of translational regulation, and to receive a quantification of the number of genes regulated at each level as a bar plot (Fig. 2e). All figures as well as a list of mRNAs selected for each type of translational regulation can be downloaded in the sidebar panel.

## Results

We used RIVET to re-analyze previously published datasets to demonstrate the it’s utility in analyzing data generated by three commonly used platforms: microarray, polysome profiling, and ribosome profiling.

### Microarray data

**Fig. 4:**
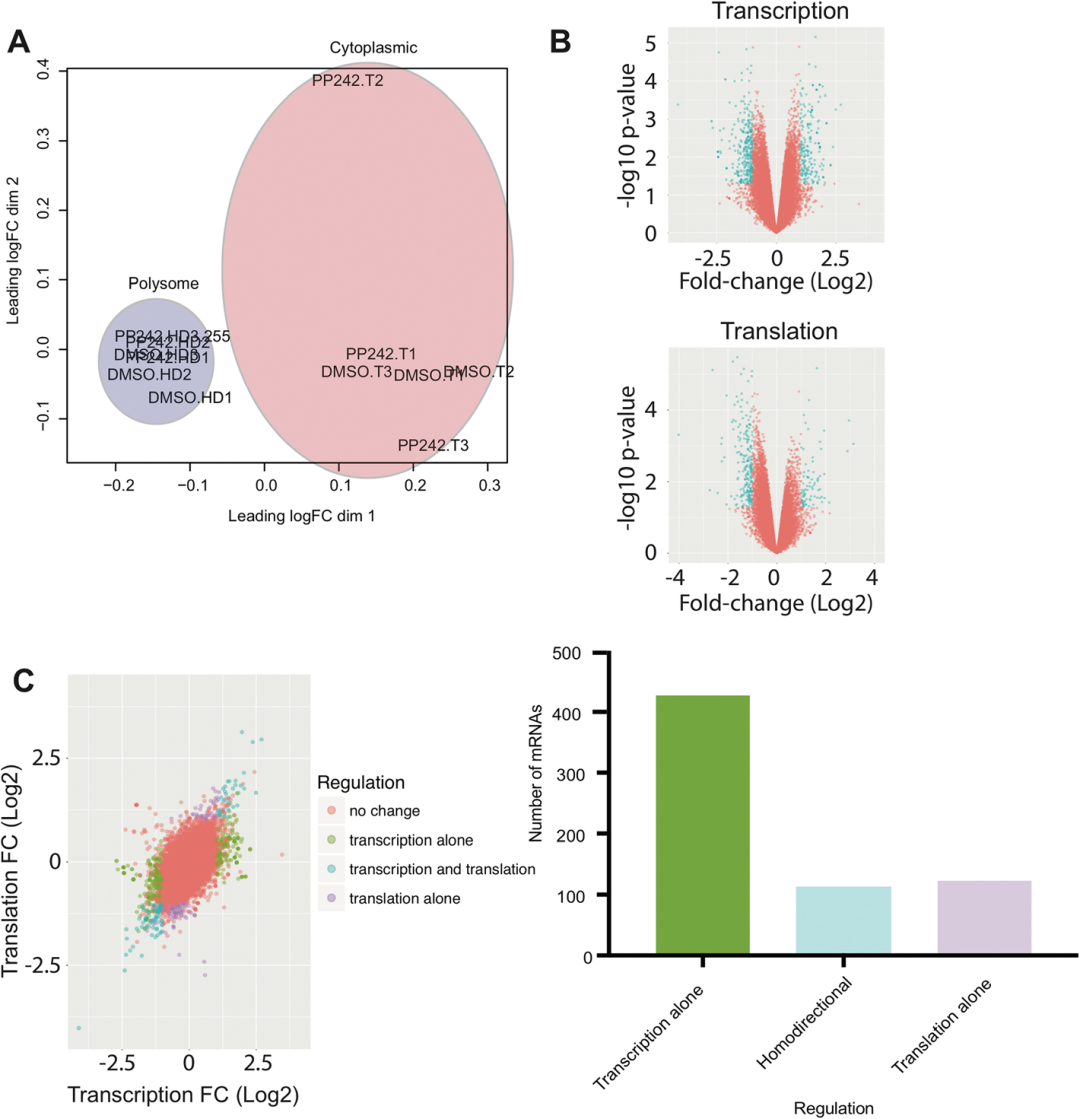
RIVET analysis of microarray polysome profiling data. **a**. MDS plot of microarray samples. Highlighted in blue are samples obtained from polysomes and in red are cytoplasmic samples. **b**. Volcano plots with significant mRNAs highlighted in blue. The transcriptional volcano plot (top) has more significant mRNAs than the translational volcano plot (bottom). **c**. Translational regulation analysis. Highlighted mRNAs were selected to display the different types of translational regulation present in the data. mRNAs in red are those not regulated. Those in green are those that were selected to be regulated in transcription but not translation. In blue are mRNAs that are both regulated at the level of transcription and translation. In purple are mRNAs that are regulated in translation alone and not transcription. Not shown are mRNAs regulated in opposite directions in transcription and translation as this type of regulation was not found in this dataset. A quantification of types of regulation is provided on the right

Data from Silvera et al. was used as a microarray analysis test set [3]. This study compared the effects of treatment with two mTOR inhibitors, PP242 and RAD001, with and without irradiation on the selective translation of mRNAs in an inflammatory breast cancer cell line. To reduce the complexity of the dataset, we used only PP242 treated samples and DMSO control samples subsampled from the spreadsheet of RMA background corrected and filtered data downloaded from GEO accession GSE92598. Data was uploaded into RIVET in the normalization module using the microarray option for downstream analysis. The RIVET-generated MDS plot revealed that the greatest separation in this dataset was between polysome-derived samples and cytoplasmic samples which can be typical of microarray-derived polysome experiments [22] (Fig. 4a). Comparison of the transcription and polysome derived volcano plots (log2 fold-change > 1; *p*-value < 0.05), PP242 effects are largely at the level of transcription compared to translation (Fig. 4b). Interestingly, when examining all types of regulation in the translational efficiency module, most genes appear to be translationally buffered; i.e. genes are regulated at the level of transcription without corresponding changes occurring in translation (Fig. 4c). Quantification of significant genes in transcription, translation, and all regulation can be handled by examining the number of genes produced in transcription and translation spreadsheets as well as counting the number of items per category in the all regulation spreadsheet.

### Polysome profiling data

**Fig. 5:**
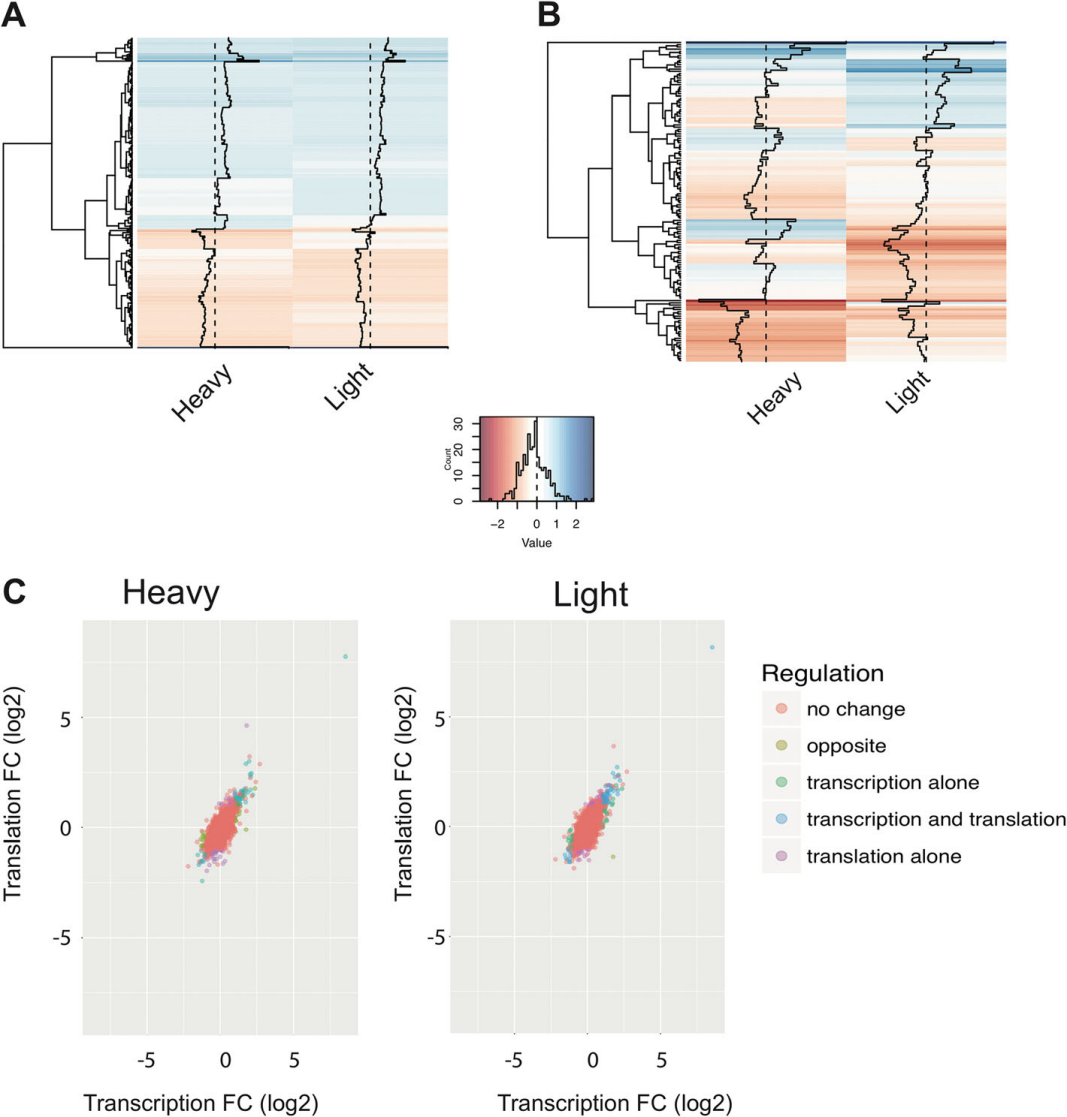
RIVET analysis of polysome profiling data with multiple polysome fractions. **a**. Heatmap from translation module, polysome sub-tab, of fold-changes of eIF4E silencing compared to control in MCF7 cells in heavy (> 4 ribosomes) or light (2–3 ribosomes). Fold-changes are either upregulated (in blue) across the gradient or downregulated (in red) **b**. Translational regulation scatter plot with types of translational regulation highlighted in different colors. The threshold for regulation was set at translation > 4 fold and transcription > 1 fold in the translation and transcription modules respectively. **c**. Heatmap of mRNAs selected to have a translational efficiency > 1 fold, *p*-value < 0.05 in either heavy or light polysome fractions. Fold-changes correspond to eIF4E silencing compared to control in polysome fractions

To demonstrate the utility of RIVET in polysome profiling, we used data generated by Geter et al. [2]. Unlike most studies, the authors of this study combined fractions 4–6 to study heavily translated mRNAs and fractions 2–3 to study poorly translated mRNAs from the polysome gradient. The goal of the study was to identify the effects of eIF4E reduction in tamoxifen-resistant breast cancer cells compared to a normal control. In total, there were two biological replicates per sample type yielding 12 samples in total. Following differential expression analysis by RIVET, a number of interesting biological observations were made. When examining eIF4E-mediated regulation occurring in translation, each mRNA was either upregulated or downregulated across the polysome gradient, which could be easily seen in the translation module with the heatmap visualization (Fig. 5a). Homodirectionality of fold-change across the polysome gradient suggests 1) many genes are regulated at the level of transcription leading to large changes that occur at the level of translation 2) changes occurring in translation are large and independent of effects in transcription.

Using RIVET, these two models can be easily verified using the translational efficiency module. With default thresholds (log2 fold change > 1; *p*-value< 0.05) for both transcription and translation, the user can discern from the all-regulation scatter plot that most changes are transcriptional and these changes are being reflected in translation. If the fold-change threshold is increased to examine the largest effect size, the scatter plot demonstrates that most genes are both transcriptionally and translationally regulated (Fig. 5c). Though transcriptional changes lead to the largest effects in translation in this dataset, as has been observed in many studies, small effects in translation can affect biology dramatically. And these small changes in translation tend to be in translationally-regulated mRNAs that can be uniquely identified using polysome profiling [5]. To examine the dynamics of this particular subset of mRNAs, the user can use both the translation and the translational efficiency module. The heatmap graphic will highlight the dynamics of each mRNA that is regulated at the level of translational efficiency across the polyribosome gradient. Immediately apparent in the heatmap are the heterodirectionality of fold-changes across the gradient, suggesting shifts in translation from heavier to lighter polyribosome fractions (Fig. 5b). Therefore, the dynamic features of RIVET allow users to gain clearer and more robust biological insight from data than would be possible using static methods.

#### Ribosome profiling data

**Fig. 6:**
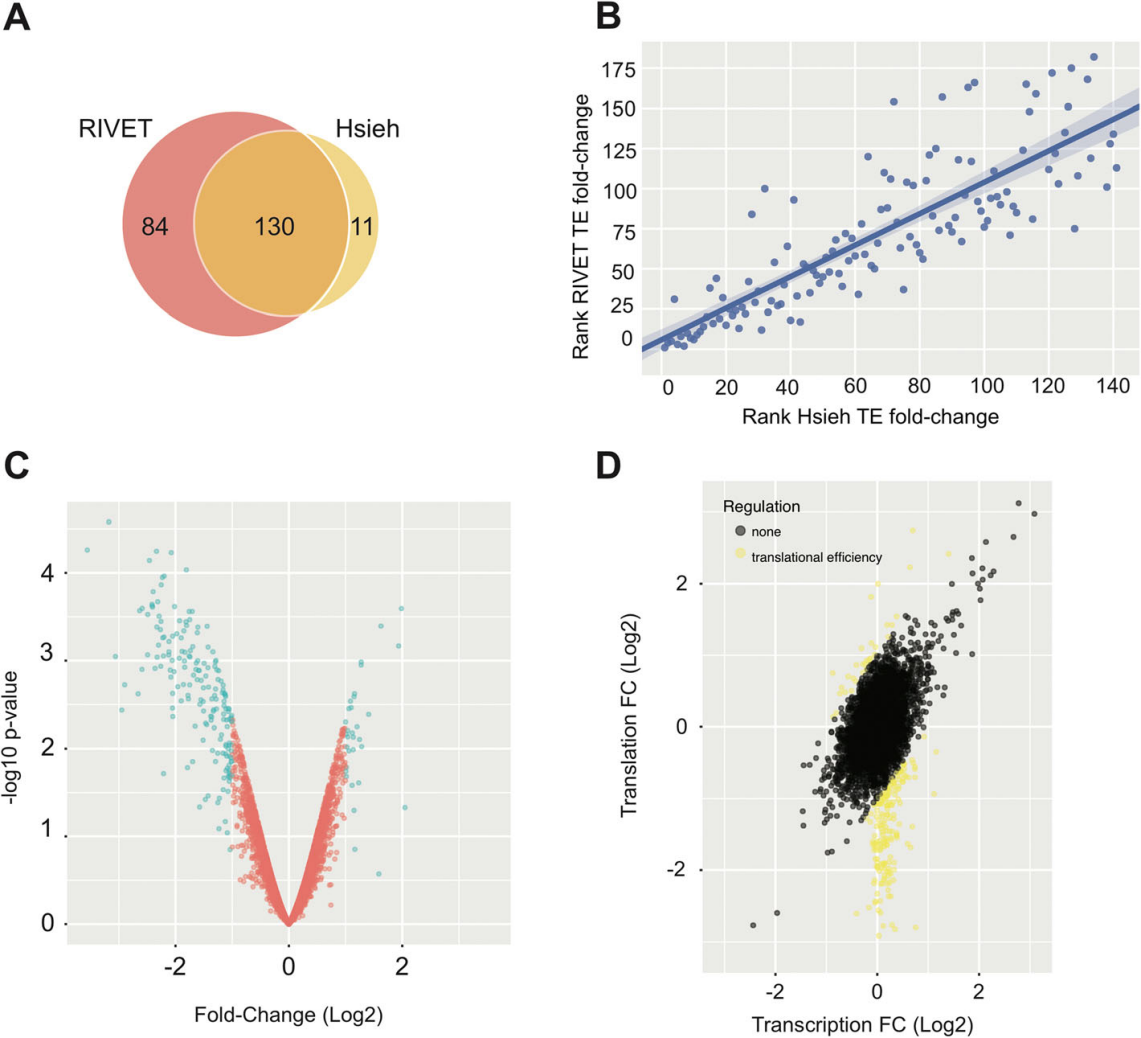
RIVET analysis of ribosome profiling data. **a**. Venn diagram depicting overlap of RIVET and Hsieh mRNA selection in translational efficiency data downloaded from Hsieh et al. mRNAs were selected in RIVET with log fold-change > 1-fold to compare to selection criteria in Hsieh et al. (log FC > 1.5-fold, FDR corrected for log fold change) **b**. Spearman correlation of ranks of mRNAs selected in Hsieh et al. and RIVET ranked by log fold-change. **c**. Volcano plots from translational efficiency module in RIVET with mRNAs selected displaying log fold-change > 1. **d**. Scatter plot from the translational regulation module displaying mRNAs selected for translation efficiency > 1-fold

Ribosome profiling data (GSE35469) were obtained from Hsieh et al. and examined the comparison of prostate cancer (PC3) cells treated with or without PP242, a drug targeting the mTORC1/2 complex [23]. The original study design implemented 2 biological replicates and we utilized PP242-treated or vehicle treated samples with paired ribosome profiling and RNA sequencing samples to give a total of 6 samples. Using downloaded counts for each sample, we analyzed 5,688 genes as input into the RIVET differential analysis pipeline after filtering for genes with greater than 256 reads in RNA sequencing samples (as per methods described in Hsieh et al.). To ascertain our ability to use RIVET to analyze ribosome footprinting data, we compared the ability of the limma statistical framework within RIVET to select consistently regulated mRNAs to those found in Hsieh et al. using a log fold-change threshold > 1.5 as described. We found that ~ 60% of translational efficient mRNAs in Hsieh et al., were also identified by RIVET (Fig. 6a). As fold-changes in Hsieh et al. overall were larger than those calculated by RIVET, we calculated a compression score for RIVET fold-changes, and transformed the 1.5-fold threshold by this value yielding a new threshold of 1-fold for RIVET data to allow for a more comparable fold-change comparison between studies. Using this fold-change setting in RIVET we were able to recapitulate 90% of translationally efficient mRNAs in Hsieh et al. and identify 84 additional mRNAs. The rank of fold-changes for translational efficiency were similar between Hsieh et al. and RIVET (rho = 0.88 Spearman correlation of rank differences) (Fig. 6b). Differences between data are likely due to differences in selection criteria between analysis as Hsieh et al. utilized a false-discovery rate correction for fold-change thresholds as opposed to *p*-values for selected mRNAs as described in supplemental Fig. 4 of their paper. Although differences exist between methodologies, RIVET successfully identifies trends described in Hsieh et al. For example, a majority of mRNAs are down-regulated in translational efficiency in agreement with Hsieh et al. (Fig. 6c). Further, when translational efficiency is examined utilizing the translational regulation module in RIVET, most mRNAs show little change at the level of transcription with robust changes in translation as concluded by the Hsieh et al. (Fig. 6d). These data suggest that RIVET can be utilized to distinguish differential translation and translational regulation in ribosome profiling data.

### Comparison to other resources

Rivet has several advantages over currently available packages that carry out differential expression analysis in ribosome profiling and polysome profiling experiments, summarized in Table 3. Like Riborex [8] and tRanslatome [24], RIVET uses existing generalized linear model (GLM) based frameworks, either limma or edgeR depending on user selection. These GLMs have been shown to complete more rapid analysis compared with similar GLM methods such as Xtail [10], RiboDiff [9], and Babel [7, 8] . Moreover, the use of limma or edgeR support the analysis of small sample size (less than 3 biological replicates) unlike anota2seq, which require 3 biological replicates unless there are more than 2 experimental conditions [17]. However, unlike all other translatome tools, RIVET makes use of a graphical user interface to make these statistical frameworks accessible to users with limited computer programming abilities. Also, unlike other tools, RIVET offers the advantage of allowing users to include analysis of multiple polysome fractions which may be critical in capturing nuances in translational regulation that have important impacts on biology. Important to the use of legacy microarray polysome profiling data, RIVET, like anota2seq [17], can analyze both continuous microarray data as well as RNA sequencing data in current polysome profiling and ribosome profiling methodologies. As an additional advantage, as a simplification for the user, RIVET explicitly chooses the appropriate normalization method for the data depending on the platform. Finally, RIVET, leveraging multiple visualization packages such as ggplot2 and heatmap.2 function from gplot, has the capability to provide high-level and interactive data visualization to allow users to gain insight from data and produce publication ready graphs. tRanslatome incorporates data visualization with statistical analysis, however, RIVET allows for the ability for users to view visualizations interactively, alleviating the need to run scripts multiple times to gain biological insight. Limitations of this software include an inability to utilize genomic location information provided in ribosome footprinting data. RIVET is intended for differential analysis; for further exploration and visualization of ribosome footprinting data at nucleotide resolution, we refer the user to RiboGalaxy, a tool dedicated to ribosome footprinting analysis with a graphical user interface [25].

**Table 3:** Comparison of RIVET with Translatome Differential Expression Tools

Software	Rivet	Xtail	Anota2seq	Babel	RiboDiff	tRanslatome	Riborex
Novel Statistical Model		x	x	x	x		
Utilizes existing statistical frameworks	x					x	x
Visualization	x					x	
GUI	x						
RNA seq & microarray	x		x			x	
Interactive	x						
Integration of all levels of analysis	x					x	x
No Programming ability	x						
Statistical Choice	x					x	x
Multiple Polysomes	x						

## Conclusion

Translatome experiments including polysome profiling and ribosome profiling have been used to explore changes in gene expression at the level of both transcription and translation. Recently developed computational tools to examine translatome experiment data provide differential analysis or visualization of data but rarely both and lack an easy-to-use graphical user interface. RIVET is comprehensive, performing differential analysis at the level of transcription, translation, and translational efficiency in combination with interactive data visualization. RIVET can handle both microarray and RNA sequencing platforms and provides the user with the ability to analyze multiple translational fractions. Utilizing the R shiny interface, RIVET provides users without programming skills the ability to analyze multi-omics data and visualize and downloadable publication quality results. To demonstrate the utility of RIVET for all types of translational regulation studies, we re-analyzed 3 studies that examined translational regulation using RNA-sequencing based ribosome footprinting and polysome profiling with multiple polysome fractions and polysome profiling with microarrays. In conclusion, RIVET is a comprehensive, adaptable and user-friendly framework for novel hypothesis generation and data exploration which will help to drive forward the field of protein translation.

## Availability and requirements

**Project name:** Ribosomal Investigation and Visualization to Evaluate Translation.

**Project home page:** https://ruggleslab.shinyapps.io/RIVET/

**Operating system:** Platform independent

**Programming Language:** R/Rshiny

**Other requirements:** None

**License:** None

**Any restrictions to use by non-academics:** None

## Additional files

Additional file 1:  **Table S1.** Example of appropriately formatted input data with genes as rows and samples as columns. (PDF 24 kb)

Additional file 2: **Figure S1.** Example data used for RIVET. A. Schematic depicting types of data generated by a polysome profile experiment with multiple polysome fractions. There are three types of experimental data: Cytoplasmic RNA (total), Light polysome RNA, and heavy polysome RNA. Each type of data has two replicates and will contain both experiment and control samples. B. Screen shot of sample upload submodule using data from Geter et al. 1) Sample labels are provided, in this case NS (control) and E4 (experiment). Note that all labels are in accepted R notation, ie labels cannot begin with a number. 2) Transcription samples are selected from the drop-down menu. In this case, samples are labeled as follows: ‘replicate_cell line_treatment_data-type’. Control and Experiment transcription samples contain 2 replicates each. 3) The user selects the number of polysome fractions to increase the number of rows provided under the Translation section. “Conclusion”) Translation samples are selected from the drop-down menu. Like transcription, translation samples contain 2 replicates each. Each row corresponds to a different polysome fraction, Heavy pertains to the top row, light to the bottom row. (PDF 1085 kb)
