# Supplementary material for: RIVET: comprehensive graphic user interface for analysis and exploration of genome-wide translatomics data
Source: BMC Genomics. 2018 Nov 8;19:809. doi: 10.1186/s12864-018-5166-z (PMC6225633; doi:10.1186/s12864-018-5166-z)
Supplement: Supplementary file 1 — Table S1. Example of appropriately formatted input data with genes as rows and samples as columns. (PDF 24 kb) [file 12864_2018_5166_MOESM1_ESM.pdf]

gene	R1_TamR_N	R2_TamR_N	R2_TamR_N	R2_TamR_N	R2_TamR_4I	R2_TamR_4I
1/2-SBSRNA	29	17	62	48	26	43
A1BG	288	191	661	408	126	339
A1BG-AS1	60	26	97	60	30	70
A1CF	1	1	5	2	1	3
A2LD1	265	138	449	342	185	435
A2M	4	19	7	24	17	8
A2ML1	11	8	11	14	7	6
A2MP1	3	6	10	7	2	21
A4GALT	789	659	877	829	771	1007
A4GNT	0	0	0	0	0	0
AA06	0	0	0	0	0	0
AAA1	0	0	0	1	0	0
AAAS	4208	3089	1226	2628	3426	1654
AACS	2178	1939	1099	1691	2052	1333
AACSP1	14	5	7	10	7	7
AADAC	0	0	1	0	0	1
AADACL2	0	0	0	0	0	0
AADACL3	0	0	1	0	0	0
AADACL4	0	0	0	0	0	0
AADAT	1148	992	1025	1038	835	957
AAGAB	5841	5835	3583	4703	7144	4162
AAK1	781	1178	883	1142	1309	996
AAMP	12820	12382	3648	9541	13368	4618
AANAT	16	12	15	14	9	26
AARS	13567	10864	3892	10176	14340	5670
AARS2	2152	2060	1822	2316	2355	1844
AARSD1	1665	1412	947	1369	1482	965
AASDH	684	479	631	587	510	603
AASDHPPT	3967	3446	2325	2748	3612	2067
AASS	67	98	104	111	122	106
AATF	4292	3708	1169	2881	4491	1729
AATK	535	639	653	718	755	910
AATK-AS1	10	8	9	13	9	15
ABAT	2308	3031	1429	2717	2606	1318
ABCA1	0	2	2	4	1	1
ABCA10	5	8	23	13	14	30
ABCA11P	132	133	297	187	92	289
ABCA12	1699	1889	1373	1769	2843	1853
ABCA13	19	13	19	24	19	16
ABCA17P	72	50	117	62	61	105
ABCA2	3618	6439	4436	6783	7204	5893
ABCA3	5772	6099	2567	5486	8723	4296
ABCA4	20	27	8	13	52	49
ABCA5	272	380	565	441	416	608
