# Supplementary material for: RIVET: comprehensive graphic user interface for analysis and exploration of genome-wide translatomics data
Source: BMC Genomics. 2018 Nov 8;19:809. doi: 10.1186/s12864-018-5166-z (PMC6225633; doi:10.1186/s12864-018-5166-z)

Additional Figure

A

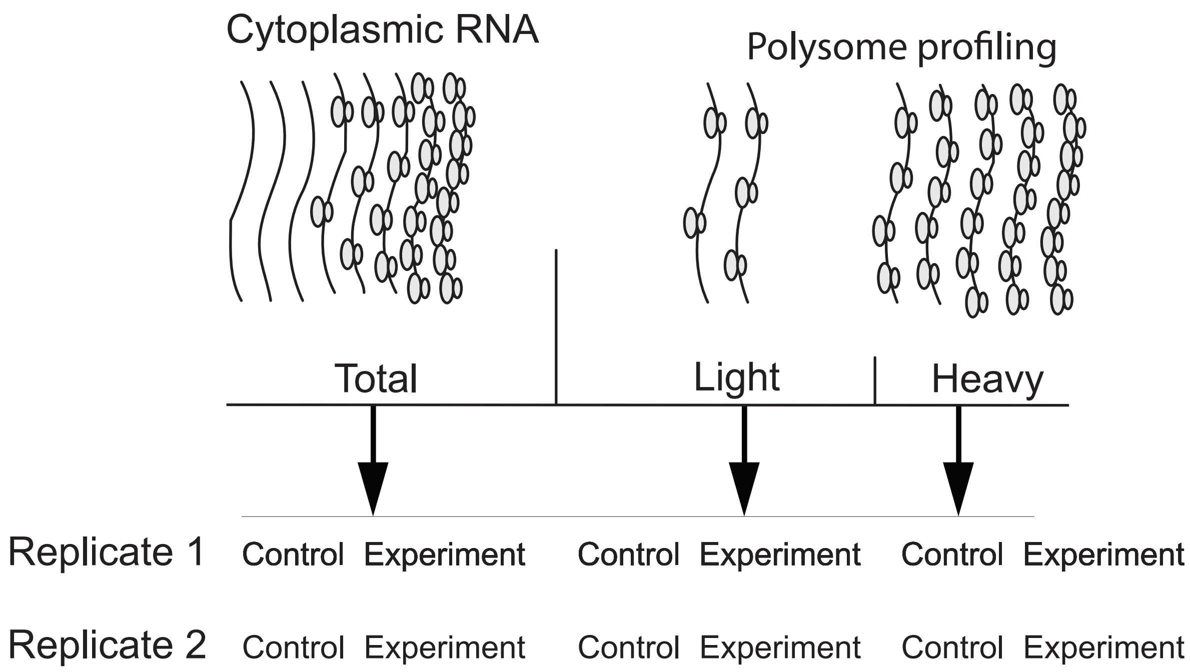

B

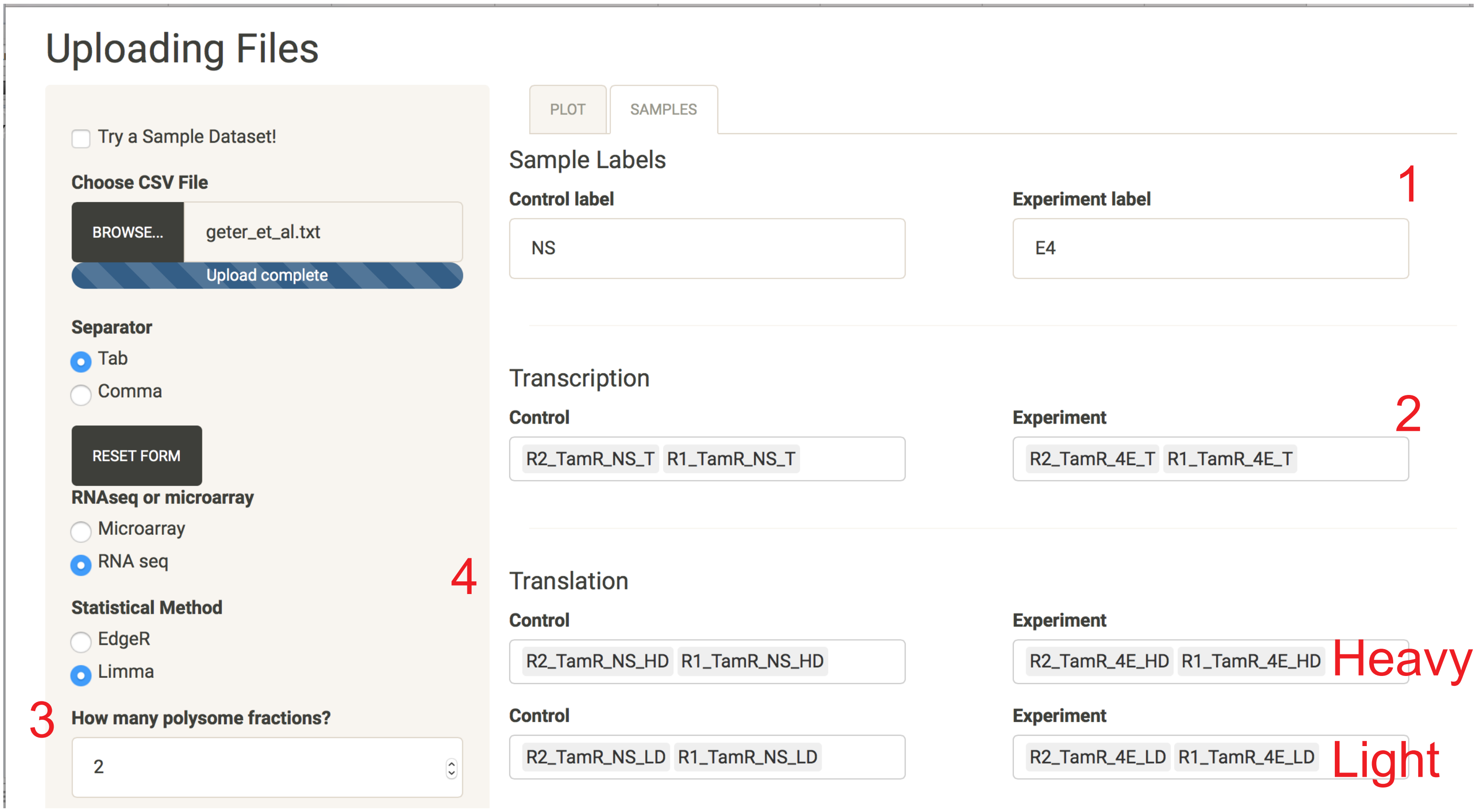

Supplement: Supplementary file 2 — Figure S1. Example data used for RIVET. A. Schematic depicting types of data generated by a polysome profile experiment with multiple polysome fractions. There are three types of experimental data: Cytoplasmic RNA (total), Light polysome RNA, and heavy polysome RNA. Each type of data has two replicates and will contain both experiment and control samples. B. Screen shot of sample upload submodule using data from Geter et al. 1) Sample labels are provided, in this case NS (control) and E4 (experiment). Note that all labels are in accepted R notation, ie labels cannot begin with a number. 2) Transcription samples are selected from the drop-down menu. In this case, samples are labeled as follows: ‘replicate_cell line_treatment_data-type’. Control and Experiment transcription samples contain 2 replicates each. 3) The user selects the number of polysome fractions to increase the number of rows provided under the Translation section. “Conclusion”) Translation samples are selected from the drop-down menu. Like transcription, translation samples contain 2 replicates each. Each row corresponds to a different polysome fraction, Heavy pertains to the top row, light to the bottom row. (PDF 1085 kb) [file 12864_2018_5166_MOESM2_ESM.pdf]
